# The Role of Sulfide Oxidation Impairment in the Pathogenesis of Primary CoQ Deficiency

**DOI:** 10.3389/fphys.2017.00525

**Published:** 2017-07-25

**Authors:** Catarina M. Quinzii, Marta Luna-Sanchez, Marcello Ziosi, Agustin Hidalgo-Gutierrez, Giulio Kleiner, Luis C. Lopez

**Affiliations:** ^1^Department of Neurology, Columbia University Medical Center New York, NY, United States; ^2^Department of Physiology, Faculty of Medicine, University of Granada Granada, Spain; ^3^MRC Mitochondrial Biology Unit Cambridge, United Kingdom

**Keywords:** coenzyme Q, CoQ, sulfides, H_2_S, sulfide:quinone oxidoreductase, SQOR

## Abstract

Coenzyme Q (CoQ) is a lipid present in all cell membranes. One of the multiple metabolic functions of CoQ is to transport electrons in the reaction catalyzed by sulfide:quinone oxidoreductase (SQOR), the first enzyme of the oxidation pathway of sulfides (hydrogen sulfide, H_2_S). Early evidence of a defect in the metabolism of H_2_S in primary CoQ deficiency came from yeast studies in *Schizosaccharomyces pombe* strains defective for *dps1* and *ppt1* (homologs of *PDSS1* and *COQ2*, respectively), which have H_2_S accumulation. Our recent studies in human skin fibroblasts and in murine models of primary CoQ deficiency show that, also in mammals, decreased CoQ levels cause impairment of H_2_S oxidation. Patient fibroblasts carrying different mutations in genes encoding proteins involved in CoQ biosynthesis show reduced SQOR activity and protein levels proportional to the levels of CoQ. In *Pdss2*^*kd*/*kd*^ mice, kidney, the only organ clinically affected, shows reduced SQOR levels and downstream enzymes, accumulation of H_2_S, and glutathione depletion. *Pdss2*^*kd*/*kd*^ mice have also low levels of thiosulfate in plasma and urine, and increased C4–C6 acylcarnitines in blood, due to inhibition of short-chain acyl-CoA dehydrogenase. Also in *Coq9*^*R*239*X*^ mice, the symptomatic organ, cerebrum, shows accumulation of H_2_S, reduced SQOR, increase in thiosulfate sulfurtransferase and sulfite oxidase, and reduction in the levels of glutathione and glutathione enzymes, leading to alteration of the biosynthetic pathways of glutamate, serotonin, and catecholamines. *Coq9*^*R*239*X*^ mice have also reduced blood pressure, possible consequence of H_2_S-induced vasorelaxation. Since liver is not clinically affected in *Pdss2* and *Coq9* mutant mice, the effects of the impairment of H_2_S oxidation in this organ were not investigated, despite its critical role in metabolism. In conclusion, *in vitro* and *in vivo* studies of CoQ deficient models provide evidence of tissue-specific H_2_S oxidation impairment, an additional pathomechanism that should be considered in the understanding and treatment of primary CoQ deficiency.

## Introduction: sulfide metabolism and mitochondria

**Figure 1:**
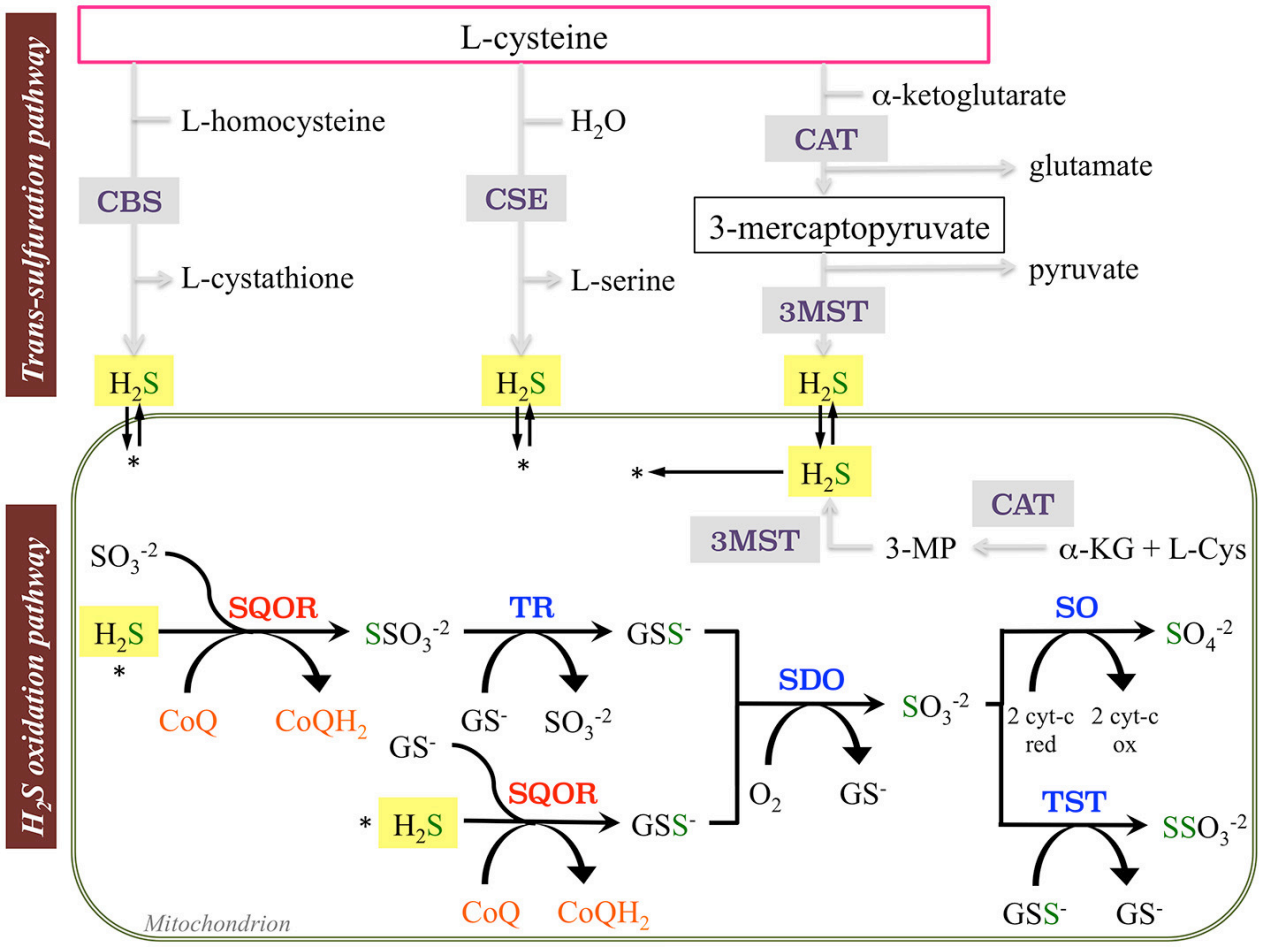
Schematic representation of sulfides (H_2_S) synthesis and oxidation pathways. The enzymes involve in the trans-sulfuration pathway are cystathionine β-synthase (CBS), cystathionine γ-lyase (CSE), and PLP-independent 3-mercaptopyruvate sulfurtransferase (3MST). The enzymes involved in the mitochondrial H_2_S oxidation pathway are sulfide:quinone oxidoreductase (SQOR), sulfur dioxygenase (SDO; also known as ETHE1 or persulfide dioxygenase), sulfite oxidase (SO), thiosulfate sulfurtransferase or rhodanese (TST), and thiosulfate reductase (TR) (Tables A1, A2).

Sulfide metabolism in mammalian cells includes the trans-sulfuration (biosynthetic) and the hydrogen sulfide (H_2_S) oxidation (catabolic) pathways. H_2_S is produced endogenously by the desulfuration of cysteine or homocysteine by the cytoplasmic enzymes cystathionine β-synthase (CBS) and cystathionine γ-lyase (CSE, CTH). H_2_S is also produced in the reaction catalyzed by the cytosolic/mitochondrial enzyme 3-mercaptopyruvate sulfurtransferase (3-MST), which uses 3-mercaptopyruvate as substrate (Kabil and Banerjee, 2014; Figure 1). Involvement of the trans-sulfuration pathway in mitochondrial pathology has been recently demonstrated. In human dopaminergic neurons, the complex I inhibitor MPP+ induces activation of branches of the trans-sulfuration pathway, mediated by the transcription factor ATF4, to increase glutathione (GSH; Krug et al., 2014). ATF4-mediated activation of serine biosynthesis and trans-sulfuration pathway was also observed in HEK-293 cells and muscle with mitochondrial DNA depletion (Bao et al., 2016; Nikkanen et al., 2016).

At least four enzymes participate to the catabolism of H_2_S in the mitochondria and sequentially perform the oxidation of the sulfide into a sulfate ion (Figure 1). The first one, sulfide:quinone oxidoreductase (SQOR), transfers sulfane sulfur atoms from H_2_S to free sulfites, and generates thiosulfate. During this reaction, electrons are shuttled from sulfide to the mitochondrial electron transport chain by reduction of ubiquinone (CoQ) to ubiquinol (CoQH_2_; Jackson et al., 2012). Then, a sulfur dioxygenase (SDO or ETHE1) converts the product of this reaction, GSH persulfide (GSSH) to sulfite, releasing GSH. Sulfite can then be oxidized to sulfate by sulfite oxidase (SO); alternatively, the thiosulfate sulfurtransferase or rhodanese (TST) converts sulfide to thiosulfate via addition of a persulfide. The sulfane sulfur from thiosulfate can be remobilized by another sulfurtransferase called thiosulfate reductase (TR) and sulfate can be secreted into the blood and eliminated through the urine (Muller et al., 2004; Hildebrandt and Grieshaber, 2008; Figure 1).

The interplay of catabolism and the upstream biosynthesis pathways probably contributes to the regulation of H_2_S levels, as suggested by recent studies in patients and an animal model of Crohn's disease (Mottawea et al., 2016). Mottawea and colleagues showed that patients with Crohn's disease have an increase in the intestinal H_2_S microbial producers with a parallel decrease in the enzymes of the H_2_S oxidation pathway (Mottawea et al., 2016). Consistently, administration of an H_2_S scavenger in mice with Crohn's disease mitigated their colitis, revealing the importance of H_2_S oxidation pathway in inflammatory bowel disease (Mottawea et al., 2016).

Hydrogen sulfide, together with nitric oxide and carbon monoxide, is a gas modulator involved in numerous physiological functions such as cell proliferation, angiogenesis, cardioprotection, neural development, prevention of oxidative stress, and apoptosis (Bouillaud and Blachier, 2011). Several lines of evidence also indicate that at concentrations of 1–10 μM, H_2_S is utilized by SQOR to maintain mitochondrial electron transport and to produce ATP in mammalian cells (Modis et al., 2013). In physiological conditions, the oxidation of H_2_S appears to contribute marginally to the cellular oxygen consumption and mitochondrial ATP synthesis, due to the major utilization of reducing equivalents through complex I. However, cells in sulfide-rich environments as colonocytes allow SQOR functioning at its maximal rate independently of the presence of other substrates and cellular ATP demand (Lagoutte et al., 2010). Albeit, if accumulated (>10 μM), H_2_S becomes toxic, causing cytochrome *c* oxidase (COX, complex IV) deficiency, by inhibiting *heme a* (Di Meo et al., 2011), and dicarboxylic aciduria, through inhibition of the enzymatic activity of short-chain acyl CoA dehydrogenase (SCAD; Pedersen et al., 2003).

Therefore, H_2_S seems to have a consistent and congruent biphasic effect in the mitochondrial respiratory chain: at over-physiological concentrations it is a COX inhibitor while at physiological concentrations it serves as mitochondrial substrate equivalent to Krebs cycle—derived electron donors—such as, NADH or FADH2.

Hydrogen sulfide also participates in the relaxation of blood vessels by opening ATP-sensitive K+ channels in vascular smooth muscle (Yang et al., 2008), in inflammatory modulation (Yang et al., 2013) and in the production of reactive oxygen species (ROS; Eghbal et al., 2004). Accumulation of H_2_S in the nervous system induces increase in the concentration of serotonin and a decrease in GABA, aspartate, norepinephrine, and glutamate (Skrajny et al., 1992; Roth et al., 1995). One mechanism of action of H_2_S is through modification of cysteine residues of target proteins by S-sulfhydration (sulfhydration, persulfhydation). Oxidative post-translational modifications of Cys residues in proteins are important for regulation of different cell functions. S-sulfhydration usually affects proteins function exerting opposite effects of nitrosylation, therefore enhancing their function (Mustafa et al., 2009; Paul and Snyder, 2012). For example, S-sulfhydration has been shown to regulate ATP5A1 (a subunit of the mitochondrial ATP synthase; Modis et al., 2016), to increase transcriptional activity of the Krupper-like factor 5 (KLF5; Meng et al., 2016), and to induce Nrf2 dissociation from Keap1, thus enhancing Nrf2 nuclear translocation (Xie et al., 2016).

## Yeast models: the first evidence of H_2_S accumulation in CoQ deficiency

In fission yeast, the enzyme sulfite reductase is responsible for the synthesis of sulfide from sulfite (Vande Weghe and Ow, 1999). Sulfide is necessary for the biosynthesis of cysteine and methionine; cysteine is synthetized by cysteine synthase from O-acetylserine and sulfide (Fujita and Takegawa, 2004), while homocysteine is synthetized by homocysteine synthase from O-acetylhomoserine and sulfide (Brzywczy et al., 2002; Fujita et al., 2006). Sulfide is oxidized by sulfide-quinone oxidoreductase encoded by *hmt2*, which was originally identified in a mutant highly sensitive to Cd^2+^ (Vande Weghe and Ow, 2001).

**Figure 2:**
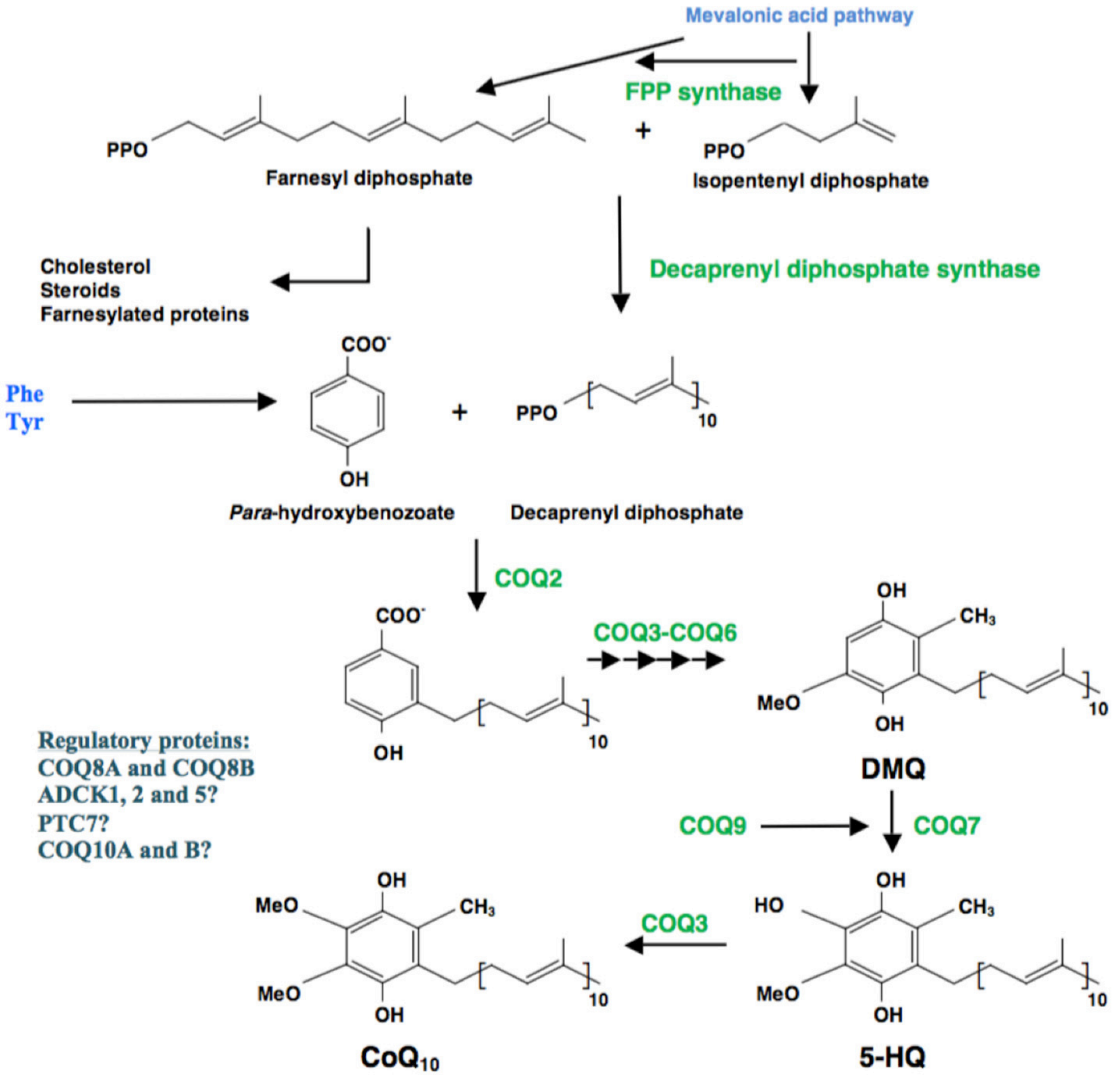
Schematic representation of CoQ biosynthesis. Coenzyme Q_10_ (CoQ_10_) is the predominant form of CoQ in humans and is synthesized in the mitochondrial inner membrane. CoQ_10_ is composed of a benzoquinone ring, derived from tyrosine or phenylalanine, and an isoprenoid side chain, synthetized in multiple steps by the enzyme decaprenyl diphosphate synthase. PHB-polyprenyl transferase (COQ2) is responsible for the condensation of decaprenyl diphosphate and para-hydroxybenzoate (PHB). The benzoate ring is then modified by at least six enzymes, which catalyze methylation, decarboxylation, and hydroxylation reactions to synthesize CoQ_10_ (Tables A1, A2).

In 2000, Uchida and colleagues characterized a strain of *Schizosaccharomyces pombe* with a defect in its PHB polyprenyltransferase gene, *ppt1 (COQ2* homolog*)*, encoding the second enzyme of the CoQ biosynthetic pathway (Figure 2), unable to produce CoQ, and accumulating H_2_S (Uchida et al., 2000). This observation suggested that if cells lack CoQ, SQOR cannot function and thus the cell accumulates H_2_S. The same phenotype was subsequently shown to be present in *S. pombe* strains disrupted for all the individual *coq* genes (Figure 2; Zhang et al., 2008; Hayashi et al., 2014). Moreover, in all tested strains grown in both, rich and minimum media, sulfide levels were lowered by addition of cysteine, suggesting that cysteine controls the production of sulfide.

## Mammalian studies *In vitro:* the H_2_S oxidation pathway is impaired in human CoQ deficiency, proportionally to the degree of CoQ deficiency and independently of the molecular defect

In mammals, CoQ is a lipid-soluble present in all cell membranes and is involved in multiple metabolic functions. One of these functions is to shuttle electrons in the first reaction of the H_2_S oxidation pathway, catalyzed by SQOR (Figure 1). Our studies in human fibroblasts confirm that low levels of CoQ cause decrease of SQOR protein levels, proportionally to the degree of CoQ deficiency (Luna-Sanchez et al., 2017; Ziosi et al., 2017). We showed that fibroblasts carrying mutations in different CoQ biosynthetic genes—*PDSS2, COQ2, COQ4, COQ8A*, and *COQ9*—have decrease of SQOR driven-respiration, and SQOR steady state protein levels, proportional to the severity of CoQ deficiency (Luna-Sanchez et al., 2017; Ziosi et al., 2017). The defects observed in COQ mutant fibroblasts are rescued by CoQ supplementation (Luna-Sanchez et al., 2017; Ziosi et al., 2017). Moreover, pharmacological inhibition of CoQ biosynthesis *via* 4-NB in wild-type fibroblasts and by COQ8A/ADCK3 depletion in HeLa cells partially recapitulate the COQ mutant cells phenotype, indicating that they are caused by CoQ deficiency (Ziosi et al., 2017). The levels of residual SQOR protein and the availability of catabolites seem to regulate the pathway downstream, since in patients and 4-NB treated fibroblasts, and in COQ8A depleted HeLa cells, SQOR protein levels are partially reduced, and the other enzymes of the pathway are increased; on the contrary, severe SQOR depletion in Hela cells shuts down the pathway (Ziosi et al., 2017).

The mRNA levels of the enzymes of the H_2_S oxidation pathway differ among CoQ deficient cell lines, suggesting that in CoQ deficiency, SQOR levels are regulated by CoQ amount at translational level; if the residual CoQ is very low, SQOR protein is degraded because unstable. However, the striking effects of CoQ synthesis inhibition and of CoQ supplementation on *SQOR* mRNA levels suggest that changes in CoQ levels modulate gene expression (Ziosi et al., 2017). There are previous evidences that CoQ affects biological processes, as lipid metabolism, inflammation, and cell signaling, through regulation of gene expression, mediated by its antioxidant function (Schmelzer et al., 2008; Fischer et al., 2016).

Sulfide accumulation in COQ mutant fibroblasts leads to increased protein S-sulfhydration, particularly targeting cytoplasmic ribosomal proteins and proteins involved in cell redox status. Further studies are needed to assess whether S-sulfhydration affects these proteins function.

## Mammalian *In vivo* studies: the connection of the disruption in sulfide metabolism and the decreased levels of glutathione as a possible pathogenic mechanism in CoQ deficiency

We investigated the tissue-specific effects of CoQ deficiency on H_2_S oxidation in three mouse models with different phenotype associated to CoQ deficiency: *Pdss2*^*kd*/*kd*^ mice, which carry a spontaneous mutation in *Pdss2*, which encodes the subunit 2 of polyprenyl-diphosphate synthase, the first enzyme of CoQ biosynthesis (Peng et al., 2004; Saiki et al., 2005), and two knock-in mice harboring mutation in *Coq9* (Garcia-Corzo et al., 2013; Luna-Sanchez et al., 2015), which encodes COQ9, a protein that interact with COQ7, the enzyme responsible for the hydroxylation of demethoxyubiquinone to 5-hydroxyquinone (Figure 2; Garcia-Corzo et al., 2013). Adult *Pdss2*^*kd*/*kd*^ mice develop nephrotic syndrome, and subsequently kidney failure (Madaio et al., 2005; Peng et al., 2008). The *Coq9*^*R*239*X*^ knock-in mice have 10–15% of residual CoQ levels in cerebrum and kidney, and 10–20% in muscle, and manifest fatal mitochondrial encephalopathy, while the *Coq9*^*Q*95*X*^ mice have 40–50% of residual CoQ in cerebrum and kidney, but 10–20% in muscle, and manifest late-onset mild mitochondrial myopathy (Garcia-Corzo et al., 2013; Luna-Sanchez et al., 2015). Lohman and colleagues previously reported that steady-state levels of SQOR were reduced in heart and kidney of *Coq9*^*R*239*X*^ mice (Lohman et al., 2014).

We observed that the protein levels of SQOR in the three mouse models studied, correlate with the level of CoQ deficiency, and affect the downstream enzymes of the H_2_S oxidation pathway (Luna-Sanchez et al., 2017; Ziosi et al., 2017). In kidney of *Pdss2*^*kd*/*kd*^ mice, which has only 15% residual CoQ, severely reduced SQOR protein levels were associated with down-regulation of all the downstream enzymes of the pathway, gluthatione (GSH) and thiosulfates reduction and mild accumulation of H_2_S, all indicative of a shut-down of the oxidation pathway. This alteration of the H_2_S oxidation pathway was also observed by severe SQOR knock down in HeLa cells, suggesting that the levels of SQOR regulate the enzymes of the down-stream pathway. In brain of *Pdss2*^*kd*/*kd*^ mice, which has ~30% residual CoQ concentrations and does not show any clinical phenotype, SQOR protein levels were slightly increased in mutant mice, and the downstream H_2_S oxidation pathway was normal (Ziosi et al., 2017).

Also in brain, kidneys and muscle of *Coq9*^*R*239*X*^ and *Coq9*^*Q*95*X*^ mice, SQOR protein levels and SQOR activity correlate with the severity of CoQ deficiency. Two months of ubiquinol-10 supplementation in *Coq9*^*R*239*X*^ mice increased muscle and kidney SQOR, proportionally to the increase of CoQ level, indicating that indeed CoQ deficiency causes the decrease of SQOR (Luna-Sanchez et al., 2017). As a consequence of the reduced SQOR levels, in *Coq9*^*R*239*X*^ mice, TST activity was increased in cerebrum and kidneys and SO levels were increased in brain. Administration of the H_2_S donor GYY4137 did not increase TST levels in wild-type mice, suggesting that the increase in TST activity is not a direct consequence of increased levels of H_2_S, but possibly of increased protein sulfhydration, caused by H_2_S accumulation. The function of proteins that can be regulated by this post-translational modification would be affected, and the expression of enzymes potentially involved in the removal of persulfide groups, such as, sulfurtransferases, might be induced.

GSH, the major non-protein thiol in cells, was decreased in affected organs of *Pdss2*^*kd*/*kd*^ and *Coq9*^*R*239*X*^ mice. In kidney of *Pdss2*^*kd*/*kd*^ GSH depletion may be caused by reactive sulfur and oxygen radical produced by H_2_S autoxidation (Truong et al., 2006), or by down-regulation of synthesis of GSH, to balance the increase of GSH caused by decrease of TST.

In cerebrum of *Coq9*^*R*239*X*^ mice, GSH depletion may be due to a decrease in the levels of glutamate, one of the three amino acids components of GSH, with the parallel increase in N-acetylglutamate, or to a reduction of its precursors, as suggested by the decreased cerebral levels of L-glutamate, an essential aminoacid for GSH biosynthesis, or to a reduction of the levels and activity of the enzymes GPx4 and GRd, which utilize GSH. These enzymes were indeed decreased, consistently with the GSH levels. This may be critical for the increase of oxidative damage previously observed in affected organs of *Pdss2*^*kd*/*kd*^ and *Coq9* mutant mice (Garcia-Corzo et al., 2013; Quinzii et al., 2013), as well as in CoQ deficient human fibroblasts, where ROS and oxidative stress levels correlate with cell viability (Lopez et al., 2010; Quinzii et al., 2010, 2012).

Since we showed that SQOR depleted Hepa1c1c7 cells have GSH levels comparable to controls (Luna-Sanchez et al., 2017), it is possible that tissue-specific abnormalities of H_2_S metabolism contribute to oxidative stress in CoQ deficiency through alteration of the GSH system. Nevertheless, we cannot exclude other factors that may be causing the low levels of GSH in CoQ deficiency.

The primary mechanism of H_2_S toxicity is the inhibition of mitochondrial complex IV (CIV; Nicholls and Kim, 1982), and mutations in the gene encoding the SDO ETHE1 causes accumulation of H_2_S in critical tissues, including colonic mucosa, liver, muscle, and brain, leading to inhibition of short-chain CoA dehydrogenase (SCAD) and CIV activities (Tiranti et al., 2004, 2009). *Pdss2*^*kd*/*kd*^ mice show increased blood levels of C4-C6 acylcarnitines, indicative of a defect of short-chain fatty acids oxidation caused by SCAD inhibition, however, surprisingly, we did not find CIV deficiency in the affected tissues of the *Pdss2*^*kd*/*kd*^ and *Coq9* mutant mice (Luna-Sanchez et al., 2017; Ziosi et al., 2017). It is possible that in CoQ deficiency H_2_S levels are not high enough to suppress CIV activity, since patients with ethylmalonic aciduria present with a much more severe phenotype associated with CIV deficiency (Tiranti et al., 2009). However, since the ethylmalonic aciduria mouse model shows normal CIV activity and level in kidney and liver, despite the high thiosulfate and H_2_S concentrations, we cannot exclude the presence of tissue-specific alternative metabolic pathways for H_2_S detoxification, or different buffering mechanisms (Tiranti et al., 2009).

## Conclusions

Several evidences *in vitro* and *in vivo* show that CoQ deficiency causes dis-regulation of the H_2_S oxidation pathway and accumulation of H_2_S that may affect multiple physiological processes, possibly through modification of protein S-sulfhydration.

Impairment of H_2_S oxidation may contribute to oxidative stress in CoQ deficiency or may play a synergistic role with oxidative stress in the pathogenesis of tissue-specificity in CoQ deficiency. The role of H_2_S metabolism defects in CoQ deficiency deserves further investigation since it may have therapeutic implications.

## Author contributions

CQ and LL: Study concept and design, acquisition of data, analysis and interpretation, critical revision of the manuscript for important intellectual content; ML, MZ, AH, and GK: Acquisition, analysis and interpretation of data, writing of the manuscript.

### Conflict of interest statement

The authors declare that the research was conducted in the absence of any commercial or financial relationships that could be construed as a potential conflict of interest.

## Appendix

**Table A1:** Abbreviations used for enzymes and other proteins.

**Abbreviation**	**Name**
SQOR	Sulfide:quinone oxidoreductase
PDSS1	Subunit 1 of polyprenyl-diphosphate synthase
PDSS2	Subunit 2 of polyprenyl-diphosphate synthase
COQ2	PHB-polyprenyl transferase
CBS	Cystathionine b-synthase
CSE	Cystathionine g-lyase
CAT	Cysteine aminotransferase
3-MST	3-Mercaptopyruvate sulfurtransferase
TST (TR)	Thiosulfate sulfurtransferase
ETHE1 (SDO)	Ethylmalonic encephalopathy protein 1 (Sulfur dioxygenase)
SUOX (SO)	Sulfide oxidase
COX	Cytochrome c oxidase
SCAD	Acyl CoA dehydrogenase
ATP5A1	Subunit A1 of the mitochondrial ATP synthase
KLF5	Krupper-like factor 5
Nrf2	Nuclear factor (erythroid-derived 2)-like 2
Keap1	Kelch like-ECH-associated protein 1
COQ8A/ADCK3	Atypical kinase COQ8A
COQ9	Ubiquinone biosynthesis protein COQ9
COQ7	5-Demethoxyubiquinone hydroxylase
COQ3	Ubiquinone biosynthesis O-methyltransferase
COQ4	Ubiquinone biosynthesis protein COQ4
COQ5	2-Methoxy-6-polyprenyl-1,4-benzoquinol methylase
COQ6	Ubiquinone biosynthesis monooxygenase COQ6

**Table A2:** Abbreviations used for substrates and products in enzymatic reactions.

**Abbreviation**	**Name**
CoQ	Coenzyme Q (ubiquinone)
H_2_S	Hydrogen sulfide
GSH	Reduced gluthatione
GSSG	Oxidized gluthatione
CoQH_2_	Reduced coenzyme Q (ubiquinol)
${\text{SO}}_{3}^{-2}$	Sulfite
${\text{SSO}}_{3}^{-2}$	Thiosulfate
${\text{SO}}_{4}^{-2}$	Sulfate
ROS	Reactive oxygen species
2 cyt-c red	Cytochrome c reduced
2 cyt-c ox	Cytochrome c oxidized
PHB	Para-hydroxybenzoate
4-NB	4-Nitrobenzoate
DMQ	Demetoxyubiquinone
5-HQ	5-Hidroxyquinone
L-Cys	L-Cysteine
α-KG	α-Ketoglutarate
3-MP	3-Mercaptopyruvate
FPP	Farnesyl Diphosphate
Phe	Phenilalanine
Tyr	Tyrosine
GPx4	Glutathione peroxidase
GRd	Glutathione reductase
